# Intrathecal heat shock protein 60 mediates neurodegeneration and demyelination in the CNS through a TLR4- and MyD88-dependent pathway

**DOI:** 10.1186/s13024-015-0003-1

**Published:** 2015-02-26

**Authors:** Karen Rosenberger, Paul Dembny, Katja Derkow, Odilo Engel, Christina Krüger, Susanne A Wolf, Helmut Kettenmann, Eckart Schott, Andreas Meisel, Seija Lehnardt

**Affiliations:** Department of Neurology, Charité-Universitaetsmedizin Berlin, Charitéplatz 1, 10117 Berlin, Germany; Center for Stroke Research, Charité-Universitaetsmedizin Berlin, Charitéplatz 1, 10117 Berlin, Germany; Cellular Neurosciences, Max Delbrueck Center for Molecular Medicine, Robert Roessle Str. 10, 13125 Berlin, Germany; Department of Hepatology and Gastroenterology, Charité-Universitaetsmedizin Berlin, Augustenburger Platz 1, 13353 Berlin, Germany; Cluster of Excellence NeuroCure, Charité-Universitaetsmedizin Berlin, Charitéplatz 1, 10117 Berlin, Germany; Institute of Cell Biology and Neurobiology, Center for Anatomy, Charité-Universitaetsmedizin Berlin, Charitéplatz 1, 10117 Berlin, Germany

**Keywords:** Neurodegeneration, Innate immunity, Heat shock protein 60, Toll-like receptor, Intrathecal injection, Cerebral ischemia

## Abstract

**Background:**

Toll-like receptors (TLR) constitute a highly conserved class of receptors through which the innate immune system responds to both pathogen- and host-derived factors. Although TLRs are involved in a wide range of central nervous system (CNS) disorders including neurodegenerative diseases, the molecular events leading from CNS injury to activation of these innate immune receptors remain elusive. The stress protein heat shock protein 60 (HSP60) released from injured cells is considered an endogenous danger signal of the immune system. In this context, the main objective of the present study was to investigate the impact of extracellular HSP60 on the brain *in vivo*.

**Results:**

We show here that HSP60 injected intrathecally causes neuronal and oligodendrocyte injury in the CNS *in vivo* through TLR4-dependent signaling. Intrathecal HSP60 results in neuronal cell death, axonal injury, loss of oligodendrocytes, and demyelination in the cerebral cortex of wild-type mice. In contrast both mice lacking TLR4 and the TLR adaptor molecule MyD88 are protected against deleterious effects induced by HSP60. In contrast to the exogenous TLR4 ligand, lipopolysaccharide, intrathecal HSP60 does not induce such a considerable inflammatory response in the brain. In the CNS, endogenous HSP60 is predominantly expressed in neurons and released during brain injury, since the cerebrospinal fluid (CSF) from animals of a mouse stroke model contains elevated levels of this stress protein compared to the CSF of sham-operated mice.

**Conclusions:**

Our data show a direct toxic effect of HSP60 towards neurons and oligodendrocytes in the CNS. The fact that these harmful effects involve TLR4 and MyD88 confirms a molecular pathway mediated by the release of endogenous TLR ligands from injured CNS cells common to many forms of brain diseases that bi-directionally links CNS injury and activation of the innate immune system to neurodegeneration and demyelination *in vivo*.

**Electronic supplementary material:**

The online version of this article (doi:10.1186/s13024-015-0003-1) contains supplementary material, which is available to authorized users.

## Background

Inflammation, neuronal injury, and demyelination are common hallmarks of most central nervous system (CNS) diseases. Innate immunity activated through Toll-like receptors (TLRs) contributes to various forms of CNS injury. TLRs are a highly conserved class of type 1 transmembrane receptors that are involved in regulation of both innate and adaptive immunity [1]. Thirteen members of the TLR family (TLR1-13) have been identified so far in humans, eleven of which are expressed in mice. Whereas some members of the TLR family including TLR3, TLR7, and TLR9 locate to intracellular compartments, other TLRs such as TLR4 are membrane-bound. TLRs detect conserved pathogen-associated molecular patterns expressed by various infectious agents including bacteria, viruses, fungi, and parasites. However, TLRs are not only activated by pathogen-associated molecules, but also by host-derived factors that are supposedly released during tissue injury [1]. For example, TLR4 is activated by both lipopolysaccharide (LPS) derived from gram-negative bacteria and host-derived molecules such as heat shock proteins (HSPs) including HSP60 and HSP70, extracellular matrix proteins, such as fibronectin extra domain A and soluble heparin sulphate, and other proteins including β-defensin and high mobility group box 1 protein [2-8].

Upon binding to their respective ligands, TLRs initiate signaling through their intracellular Toll/interleukin-1 (IL-1) receptor (TIR) domains. These interact with other TIR domains such that, upon activation, each TLR binds to a specific set of adaptor proteins that also contain TIR domains. Myeloid differentiation factor 88 (MyD88) is the universal intracellular adaptor recruited by all known TLRs except TLR3. In general, triggering of the well-described complex TLR-associated signaling pathway results in activation of various downstream effectors including NF-κB, in turn leading to an inflammatory response [1] or, occasionally, to cell death [9]. TLRs are widely expressed in innate immune cells such as macrophages and microglia in the CNS and dendritic cells, but also in non-immune cells such as neurons and epithelial cells [10,11].

Injured tissues release factors including HSPs, which represent a collection of evolutionarily conserved proteins induced in response to cellular stress such as heat shock, nutrient deprivation, or mechanical damage, and which are considered endogenous danger signals to the immune system [12-14]. In addition to serving as a cellular chaperone, extracellular HSP60 directly activates immune cells including macrophages and dendritic cells through binding to TLRs and several other membrane receptors such as CD14 [15,16]. In line with this finding, we observed in previous work that HSP60 provides a signal to microglia, the major immune cell of the brain, alerting to the presence of CNS injury through activation of a TLR4-dependent pathway in cultured CNS cells [17].

In this study we systematically analyze the impact of extracellular HSP60 on the brain. Intrathecal HSP60 results in CNS injury including neurodegeneration and demyelination. These effects require both TLR4 and MyD88. In contrast to intrathecal application of HSP60, intrathecal injection of the established pathogen-derived TLR4 ligand LPS does not affect neuronal or oligodendrocyte survival *in vivo*. However, whereas intrathecal LPS leads to robust production of proinflammatory molecules in the brain, CNS injury induced by HSP60 is not accompanied by such an inflammatory response. HSP60 is predominantly expressed in injured neurons and oligodendrocytes and is released during CNS injury such as focal cerebral ischemia *in vivo*. Based on our findings we speculate that activation of the TLR signaling pathway triggered by endogenous ligands may serve as a damage-specific danger signal in the CNS.

## Results

### TLR4 expressed in microglia is required for neuronal injury induced by HSP60

**Figure 1:**
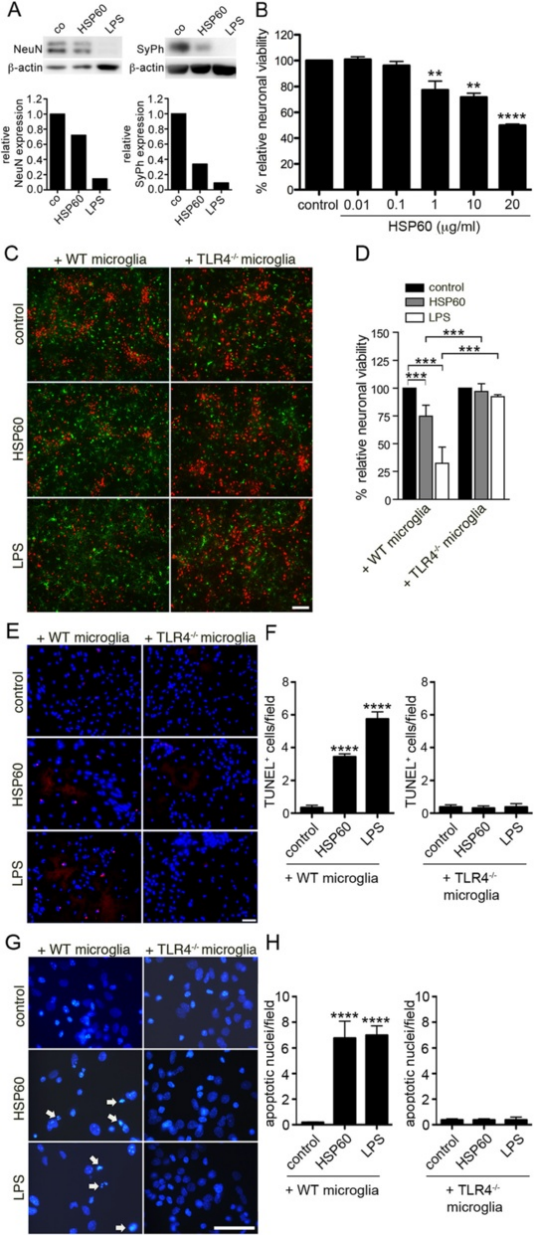
**Neurons in the presence of microglia lacking TLR4 are protected against HSP60**-**induced injury** ***in vitro*** **.** **(A)** Lysates of co-cultures containing C57BL/6 J neurons and microglia incubated with 10 μg/ml HSP60, 10 μg/ml LPS, or PBS (control) for 3 d were analyzed by SDS-PAGE and immunoblotting with neuronal nuclei (NeuN) and synaptophysin (SyPh) antibodies. β-actin served as a loading control. Immunoblot signals were analyzed by densitometry and expressed as fold change to control. One representative experiment of three independent experiments is shown. **(B)** Neurons supplemented with C57BL/6 J microglia were incubated with various doses of HSP60 or PBS (control) for 3 d. Subsequently, cells were stained with NeuN Ab. NeuN+ cells were quantified. Results were statistically expressed as relative neuronal viability by setting the viability of control cells to 100%. **(C-** **H)** Neurons supplemented with C57BL/6 J (WT) or TLR4^−/−^ microglia were incubated with 10 μg/ml HSP60 or PBS (control) for 3 d, as indicated. 1 μg/ml LPS served as a positive control. **(C)** Co-cultures were stained with NeuN antibody (red) and with IB4 (green) to mark neurons and microglia, respectively. **(D)** NeuN+ cells were quantified. Results were statistically expressed as relative neuronal viability. **(E)** Co-cultures were stained with TUNEL assay (red) and **(E,** **G)** DAPI (blue). Scale bar, 100 μm. **(F)** Quantification of TUNEL+ cells/field in cultures displayed in **E**. **(H)** Quantification of DAPI+ nuclei displaying apoptotic hallmarks, as indicated by arrows in cultures shown in **G**. **(B,** **D,** **F,** **H)** Results are presented as mean of 3–4 individual experiments, each condition performed in duplicate +/− SD. **p < 0.01, ***p < 0.001, ****p < 0.0001 (comparison of HSP60-treated groups with control in **B**; comparison of indicated groups in **D**; comparison of HSP60- and LPS-treated groups with control in **F** and **H**; two-way ANOVA with Bonferroni-selected pairs).

We observed previously that the quantity of viable neurons recovered from forebrains of mutant lps^d^ mice, in which the TLR4 signaling cascade is disrupted as the result of a naturally occurring mutation, is increased. In this *in vitro* context, HSP60 released from injured CNS cells was identified as an endogenous activator of the TLR4 signaling pathway in microglia, thereby initiating an inflammatory response and subsequent neuronal injury [17]. As confirmed by SDS-PAGE followed by immunoblotting using antibodies against neuronal nuclei and synaptophysin, HSP60 induced neurotoxic effects in co-cultures of cortical neurons from C57BL/6 J mice in the presence of microglia from C57BL/6 J mice (Figure 1A). These effects were dose-dependent, as determined by quantification of NeuN-positive cells (Figure 1B). In detail, 1 μg/ml HSP60 reduced the relative neuronal viability significantly by 22.69% (+/−6.16), 10 μg/ml HSP60 by 28.20% (+/−1.81), and 20 μg/ml HSP60 by 50.08% (+/−0.88) compared to control conditions.

To analyze the role of the microglial receptor TLR4 itself in neuronal injury induced by HSP60, co-cultures of neurons from cortices of C57BL/6 J mice in the presence of microglia from C57BL/6 J (wild-type, WT) mice or TLR4-deficient (TLR4^−/−^) mice were incubated with 10 μg/ml HSP60. While 1 μg/ml LPS served as a positive control for microglia-induced neuronal injury in this experimental set-up [18], PBS was used as a volume control. Subsequently, cell cultures were immunostained with antibodies against neuronal nuclei (NeuN) and IB4 to label neurons and microglia, respectively (Figure 1C). In cultures supplemented with C57BL/6 J microglia, incubation with HSP60 led to a significant loss of neurons. In contrast, neurons in co-cultures containing microglia lacking TLR4 were not affected by incubation with HSP60 compared with control conditions. In cell cultures supplemented with WT microglia, LPS reduced neuronal numbers to a greater extent than HSP60, as expected [17]. Quantification of NeuN-positive cells confirmed these results (Figure 1D). Increased numbers of TUNEL-positive cells (Figure 1E) and DAPI-stained nuclei displaying apoptotic hallmarks such as shrinkage and fragmentation (Figure 1G) in co-cultures containing WT microglia but not in co-cultures supplemented with TLR4^−/−^ microglia treated with HSP60 confirmed toxic effects induced by HSP60 through TLR4 *in vitro* (Figure 1F, H). Cultured neurons in the absence of microglia were not affected by HSP60 treatment (data not shown), as published before [17]. Notably, the recombinant HSP60 probe used in this approach was rigorously tested in terms of LPS contamination (see *Materials and Methods* and *Discussion*), and we excluded the possibility of LPS-related effects in HSP60-induced neuronal injury in previous work [17].

In summary, neuronal cell death induced by HSP60 requires the expression of TLR4 in microglia *in vitro*.

### Intrathecal administration of HSP60 leads to neurodegeneration in the CNS

**Figure 2:**
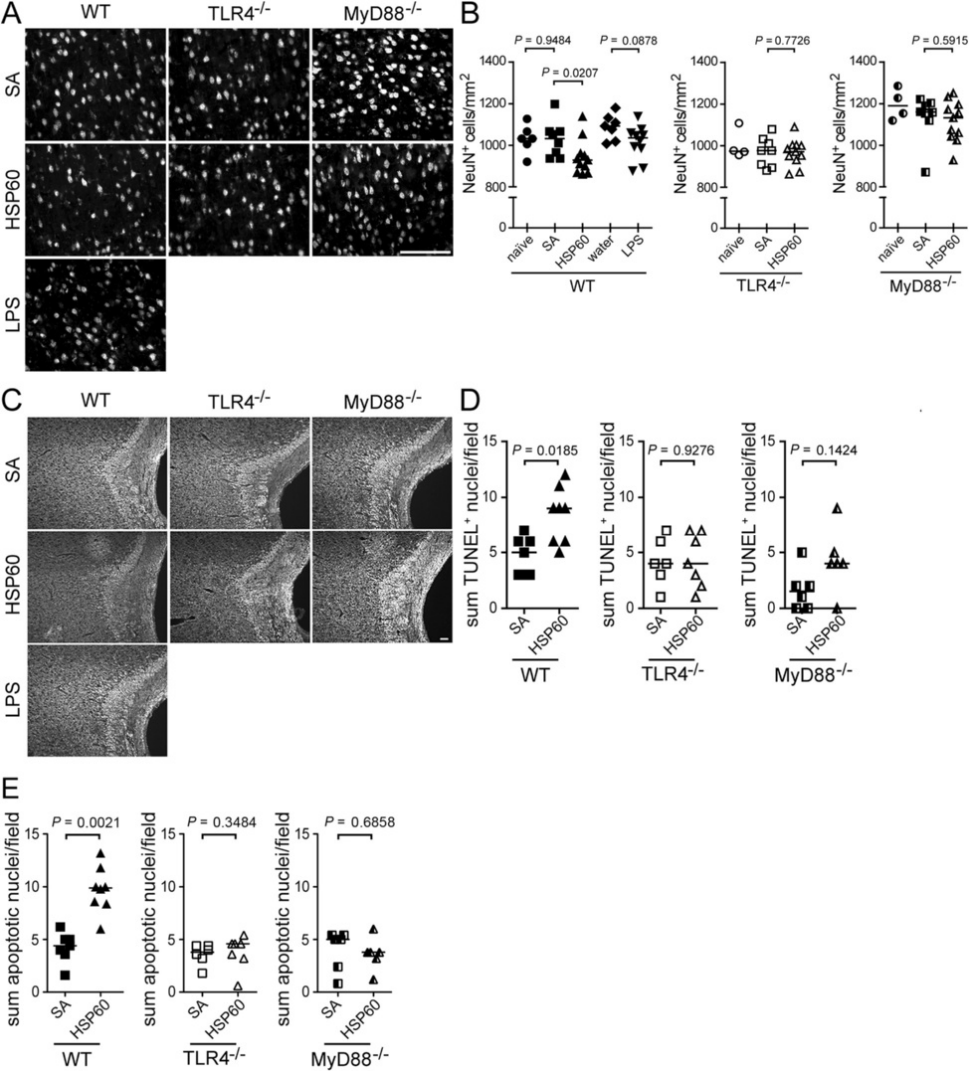
**HSP60 triggers neurodegeneration in the cerebral cortex dependent on TLR4 and MyD88** ***in vivo*** **.** **(A)** Forty micrograms of HSP60 or 40 μg of SA were injected intrathecally into 8–12 week-old C57BL/6 J (WT, HSP60 *n* = 11, SA *n* = 8), TLR4^−/−^ (HSP60 *n* = 11, SA *n* = 8), and MyD88^−/−^ (HSP60 *n* = 11, SA *n* = 8) mice. Naïve mice received no injection (WT *n* = 6, TLR4^−/−^ *n* = 4, MyD88^−/−^ *n* = 4). Injection of LPS into WT mice served as a positive control for TLR4 activation (*n* = 10). Injection of water into WT mice served as a carrier control for LPS, HSP60, and SA injection (*n* = 7). After 3 d, the cerebral cortex was analyzed by immunostaining with NeuN antibody. Scale bar, 100 μm. **(B)** Quantification of cortical NeuN+ cells of WT, TLR4^−/−^, and MyD88^−/−^ mice injected intrathecally, as described above. Median, Mann–Whitney *U* test for indicated groups. **(C)** Brain sections containing the corpus callosum of WT, TLR4^−/−^, and MyD88^−/−^ mice injected as described above were immunostained with a neurofilament antibody. Scale bar, 50 μm. Quantification of TUNEL+ cells **(D)** and DAPI-stained nuclei displaying apoptotic hallmarks including irregular shape, shrinkage, and fragmentation **(E)** in representative sections of the cerebral cortex of WT, TLR4^−/−^, and MyD88^−/−^ mice injected intrathecally with HSP60 or SA, as indicated. **(D, E)** Median, Mann–Whitney *U* test for indicated groups.

In the context of tissue injury, HSP60 is considered to serve as a danger signal for the organism because of its ability to induce a proinflammatory phenotype in innate immune cells [19]. We aimed at determining the impact of extracellular HSP60 on the brain *in vivo*. To this end, C57BL/6 J mice (WT) were injected with recombinant HSP60 intrathecally. Serum albumin (SA), a protein with a similar molecular weight as HSP60, served as a negative control. Both HSP60 and SA were solved in water. While LPS, the well-described exogenous ligand for TLR4 and also solved in water, was used for comparative analysis [20-22], water alone was used as a further control condition. No mortality of injected animals was observed over 3 days. Immunohistochemical analysis of the cerebral cortex after 3 days revealed a reduction of NeuN-positive cells in the cerebral cortex of nine out of eleven HSP60-injected animals compared to control conditions (Figure 2A). Quantification revealed a significant loss of 10.1% NeuN-positive cells in the cerebral cortex of mice injected with HSP60 compared to the SA-treated control group (Figure 2B). Stereological analysis of NeuN-positive objects and the percentage area of NeuN-positive region of interest (ROI) confirmed these results (Additional file 1: Figure S1A, B). In addition, eight out of eleven of the HSP60-treated mice displayed a distinguishable loss of axons in the corpus callosum and underlying structures (Figure 2C). In contrast, neither intrathecal injection of LPS nor injection of SA resulted in significantly reduced numbers of neurons or axonal injury compared to the carrier (water) control group or naive animals, respectively (Figure 2A-C).

To analyze whether the injurious effects induced by intrathecal HSP60 are associated with apoptosis in the CNS *in vivo*, brain sections were stained by TUNEL. Quantification of TUNEL-positive cells in four representative brain sections per brain revealed a small (1.8-fold), but significant increase of apoptotic cells in the cerebral cortex after application of HSP60 compared to treatment with SA (Figure 2D), as confirmed by stereological analysis (Additional file 1: Figure S1C, D). In accordance with these findings, quantification of DAPI-stained nuclei exhibiting morphological hallmarks of apoptosis including shrinkage and fragmentation in the cerebral cortex yielded a significant increase of apoptotic nuclei in response to intrathecal HSP60 compared to control conditions (Figure 2E).

Taken together, intrathecal HSP60 mediates neurodegeneration in the cerebral cortex.

### Neuronal injury in the CNS after intrathecal injection of HSP60 requires expression of TLR4 and MyD88

HSP60 signals through TLR4 in microglia and peripheral immune cells *in vitro* [1,17]. To test whether TLR4 signaling is involved in neurodegeneration induced by HSP60 *in vivo*, TLR4^−/−^ mice were injected intrathecally, as described above, and compared with HSP60-injected C57BL/6 J (wild-type, WT) animals. No mortality was observed in WT and TLR4^−/−^ mice over 3 days. Immunohistochemical analysis revealed that in contrast to WT mice, numbers of cortical neurons of TLR4^−/−^ mice did not differ between the HSP60-injected animals and the control groups (Figure 2A, B; Additional file 1: Figure S1A, B). In accordance with these findings, immunostaining of brain sections from TLR4^−/−^ mice with an antibody against neurofilament revealed no significant injury or loss of axonal structures in six out of seven TLR4^−/−^ mice after intrathecal application of HSP60 compared to mice treated with SA (Figure 2C). Also, in contrast to the increased numbers of TUNEL-positive cells in WT animals described above, numbers of TUNEL-positive cells in brains of TLR4^−/−^ mice were unchanged after injection of HSP60 compared to control conditions (Figure 2D; Additional file 1: Figure S1C, D).

The role of the TLR adapter MyD88 in HSP60-induced neurodegeneration *in vivo* was investigated by injecting HSP60 into MyD88^−/−^ mice, as described above, and compared with HSP60-injected C57BL/6 J (WT) animals. No mortality was observed in MyD88^−/−^ and WT mice over 3 days. In contrast to WT mice, mice lacking MyD88 were not significantly affected by injection of HSP60 regarding neuronal survival in the cerebral cortex (Figure 2A, B; Additional file 1: Figure S1A, B). Numbers of cortical neurons of MyD88^−/−^ mice injected with HSP60 were significantly higher than the neuronal numbers of WT animals injected with HSP60 (*p* < 0.05). Although a trend of enhanced TUNEL positivity in MyD88^−/−^ mice after HSP60 treatment was observed (Figure 2D), numbers of TUNEL-positive cells did not differ significantly between MyD88^−/−^ mice that received intrathecal HSP60 and the respective control animals (Figure 2D; Additional file 1: Figure S1C, D). Also, axonal structures within the cerebral cortex of MyD88^−/−^ mice were not affected by intrathecal HSP60 compared with control conditions (Figure 2C).

In accordance with these findings, quantification of DAPI-stained nuclei in the cerebral cortex of TLR4^−/−^ and MyD88^−/−^ mice revealed no differences in numbers of nuclei displaying morphological hallmarks of apoptosis between animals injected with HSP60 intrathecally and control animals (Figure 2E).

In summary, neurodegeneration following intrathecal injection of HSP60 requires TLR4 and MyD88.

### CNS injury following intrathecal injection of HSP60 includes demyelination dependent on TLR4 and MyD88

**Figure 3:**
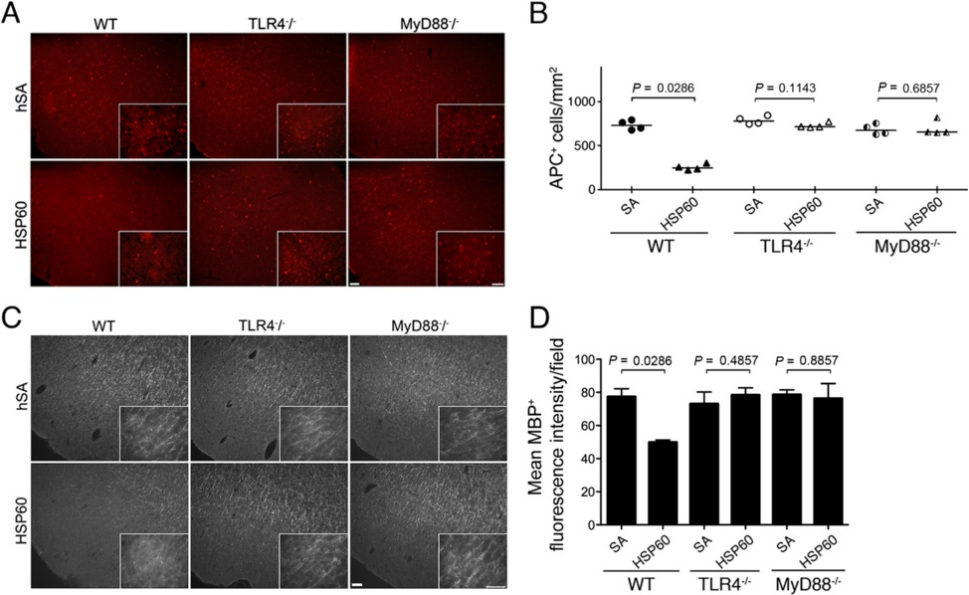
**HSP60 induces demyelination dependent on TLR4 and MyD88** ***in vivo*** **.** Three days after intrathecal injection of 40 μg HSP60 or 40 μg SA (control) coronal brain sections of C57BL/6 J (WT, HSP60 *n* = 4, SA *n* = 4), TLR4^−/−^ (HSP60 *n* = 4, SA *n* = 4), and MyD88^−/−^ (HSP60 *n* = 4, SA *n* = 4) mice were evaluated for the myelination status. **(A)** Immunostaining of the cerebral cortex of WT, TLR4^−/−^, and MyD88^−/−^ mice with an antibody directed against APC. Scale bar, 50 μm. Cut-outs at higher power from the micrographs. Scale bar, 50 μm. **(B)** Quantification of APC+ cells in the cerebral cortex of WT, TLR4^−/−^, and MyD88^−/−^ mice. Median; *p* values were determined for indicated groups by Mann–Whitney *U* test. **(C)** Immunostaining of the cerebral cortex of injected WT, TLR4^−/−^, and MyD88^−/−^ mice with an antibody against MBP. Scale bar, 50 μm. Cut-outs at higher power from the micrographs. Scale bar, 50 μm. **(D)** Analysis of mean MBP+ fluorescence intensity in the cerebral cortex of WT, TLR4^−/−^, and MyD88^−/−^ mice. Median, Mann–Whitney *U* test for indicated groups.

Our previous studies had revealed that activation of TLR4 by LPS results in oligodendrocyte injury *in vitro* [23]. To test the ability of the endogenous ligand HSP60 to induce injury of oligodendrocytes and/or myelin *in vivo*, we analyzed brain sections of mice injected with HSP60 or SA intrathecally, as described above, by immunohistochemistry using antibodies directed against adenomatous polyposis coli (APC) and myelin basic protein (MBP) to mark oligodendrocytes and myelin, respectively (Figure 3A, C). All animals from the C57BL/6 J strain treated with HSP60 showed a major loss of oligodendrocytes in the cerebral cortex compared to mice injected with SA (Figure 3A). Quantification of oligodendrocytes confirmed these results (loss of 65.34% APC-positive cells, Figure 3B and Additional file 1: Figure S1E, F). While sections from SA-injected animals after 3 days displayed a uniform abundant distribution of MBP in the cerebral cortex, injection of HSP60 resulted in a reduced immunoreactivity against MBP in all treated animals (Figure 3C), as confirmed by analysis of the mean MBP-positive fluorescence intensity (Figure 3D).

To confirm the role of TLR4 and MyD88 in oligodendrocyte injury and demyelination induced by HSP60 *in vivo*, TLR4^−/−^ and MyD88^−/−^ mice were included in the experiments described above (Figure 3A-D). None of the TLR4^−/−^ and MyD88^−/−^ mice that received HSP60 showed loss of oligodendrocytes or damage of myelin-positive structures compared to animals that received SA (Figure 3A, C). Quantitative analysis of the surviving oligodendrocytes (APC-positive cells, Figure 3B and Additional file 1: Figure S1E, F) and assessment of the mean MBP-positive fluorescence intensity (Figure 3D) in the cerebral cortex of TLR4^−/−^ and MyD88^−/−^ mice confirmed these results. Also, total numbers of APC-positive cells in the cerebral cortex of TLR4^−/−^ and MyD88^−/−^ mice treated with HSP60 were increased compared with numbers in WT animals injected with HSP60 (Figure 3B and Additional file 1: Figure S1E, F).

These data prove a toxic effect of intrathecal HSP60 towards oligodendrocytes and myelin that requires a functional TLR4 and MyD88 pathway *in vivo*.

### HSP60 mediates loss of oligodendrocyte precursor cells dependent on TLR4 expressed in microglia *in vitro*

**Figure 4:**
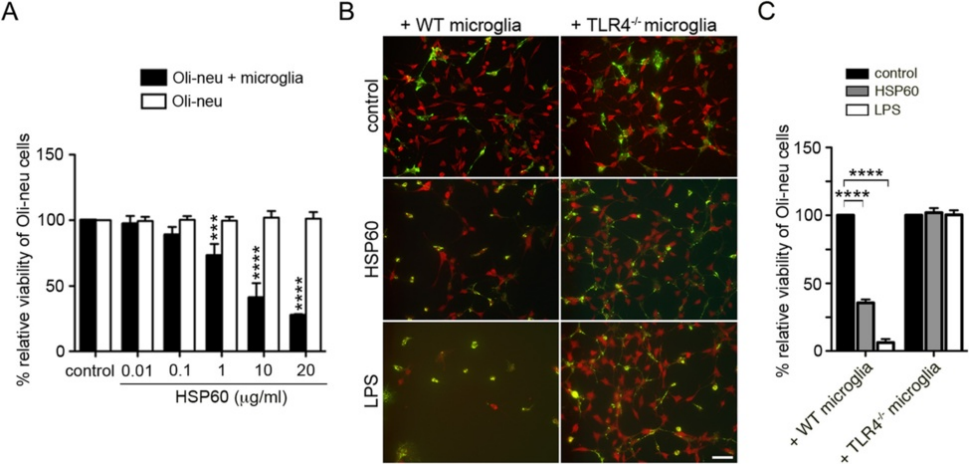
**HSP60 induces loss of Oli**-**neu cells in the presence of microglia expressing TLR4** ***in vitro*** **.** **(A)** Oli-neu cells alone or supplemented with neonatal microglia from C57BL/6 J mice were incubated with various doses of HSP60 or PBS (control), as indicated, for 3 d. Subsequently, cell cultures were stained with APC antibody to mark oligodendrocyte precursor cells. APC+ cells were quantified, and results were statistically expressed as relative viability of Oli-neu cells by setting the viability of control cells to 100%. **(B)** Oli-neu cells co-cultured with neonatal microglia derived from C57BL/6 J (WT) or TLR4^−/−^ mice were incubated with 10 μg/ml HSP60 or PBS (control), as indicated. 1 μg/ml LPS served as a positive control. After 3 d, co-cultures were fixed and stained with both APC antibody (red) and with IB4 (green) to mark oligodendrocyte precursor cells and microglia, respectively. Scale bar, 100 μm. **(C)** APC+ cells were quantified, and results were statistically expressed as relative viability of Oli-neu cells by setting the viability of control cells to 100%. **(A, C)** Results are presented as mean of 3 individual experiments with each condition performed in duplicate +/− SD. ***p < 0.001; ****p < 0.0001 (for the comparison of HSP60-treated groups with the control group in **A**; for the comparison of indicated groups in **C**; two-way ANOVA with Bonferroni-selected pairs).

To further investigate injurious effects of HSP60 on oligodendrocyte precursor cells *in vitro,* cells of the oligodendrocyte precursor line Oli-neu alone or in the presence of microglia were incubated with increasing concentrations of HSP60 for 3 days. Subsequently, cells were immunostained with an APC antibody to mark Oli-neu cells. Quantification of APC-positive cells co-cultured with microglia revealed a dose-dependent reduction of the relative viability of Oli-Neu cells induced by HSP60 (Figure 4A). In contrast, incubation of Oli-neu cells alone with HSP60 did not affect relative oligodendrocyte viability compared with control conditions, even when HSP60 was used at the highest concentration of 20 μg/ml (Figure 4A). To analyze the role of TLR4 in oligodendrocyte precursor injury induced by HSP60, co-cultures of Oli-neu cells in the presence of microglia from C57BL/6 J (WT) mice or TLR4-deficient (TLR4^−/−^) mice were incubated with 10 μg/ml HSP60. 1 μg/ml LPS served as a positive control for microglia-induced injury of oligodendrocyte precursors in this experimental set-up [23]. Subsequently, cell cultures were immunostained with APC antibody and IB4 to label Oli-neu cells and microglia, respectively (Figure 4B). In cultures supplemented with WT microglia, incubation with HSP60 led to a significant loss of Oli-neu cells. In contrast, Oli-neu cells in co-cultures containing microglia lacking TLR4 were not affected by incubation with HSP60 compared to control conditions. In cell cultures supplemented with WT microglia, LPS also reduced numbers of Oli-neu cells, as expected [23]. Quantification of APC-positive cells confirmed these results (Figure 4C).

Taken together, extracellularly applied HSP60 leads to injurious effects in oligodendrocyte precursor cells dependent on TLR4 expressed in microglia *in vitro*.

### HSP60 does not result in major glial activation or proliferation in the cerebral cortex

**Figure 5:**
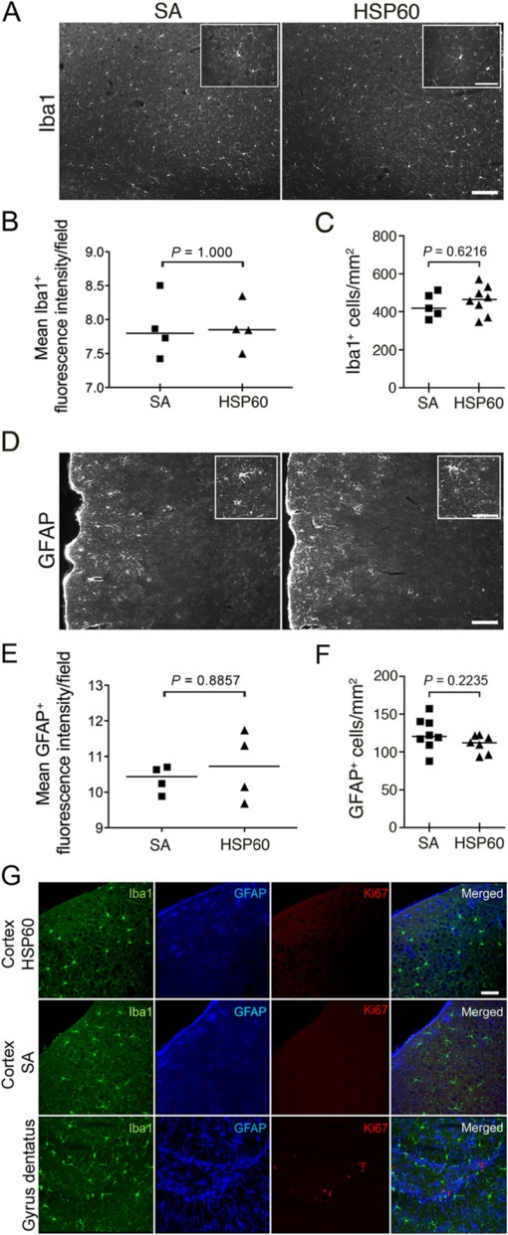
**Intrathecal HSP60 does not result in major glial activation or proliferation in the cerebral cortex** **.** Three days after intrathecal injection of 40 μg HSP60 or 40 μg SA (control) coronal sections of C67BL/6 J mouse brains were evaluated in terms of glial morphology and numbers. **(A)** Immunostaining of the cerebral cortex of C67BL/6 J mice (HSP60 *n* = 8, SA *n* = 5) with an Iba1 antibody. Scale bar, 100 μm, insets 50 μm. **(B)** Measurement of mean Iba1+ fluorescence intensity (HSP60 *n* = 4, SA *n* = 4). **(C)** Quantification of Iba1+ cells (HSP60 *n* = 8, SA *n* = 5). **(B, C)** Median, Mann–Whitney *U* test for the comparison of the HSP60-injected group with the SA-treated (control) group. **(D)** Immunostaining of the cerebral cortex of C67BL/6 J mice (HSP60 *n* = 7, SA *n* = 8) with a GFAP antibody. Scale bar, 100 μm, insets 50 μm. **(E)** Measurement of mean GFAP+ fluorescence intensity (HSP60 *n* = 4, SA *n* = 4). **(F)** Quantification of GFAP+ cells (HSP60 *n* = 7, SA *n* = 8). **(E, F)** Median, Mann–Whitney *U* test for the comparison of the HSP60-injected group with the SA-treated (control group). **(G)** Representative micrographs of the cerebral cortex immunostained with Iba1, GFAP, and Ki67 antibodies to mark microglia, astrocytes, and proliferating cells, respectively (HSP60 *n* = 4, SA *n* = 4). The subgranular zone of the hippocampal dentate gyrus served as a positive control for Ki67 reactivity. Scale bar, 100 μm.

Glial cells, in particular microglia and astrocytes, contribute to various forms of CNS injury [24]. To gain further insight into the impact of glia on the response to intrathecal HSP60 *in vivo*, brain sections from C57BL/6 J mice injected intrathecally with HSP60 or SA were immunostained with antibodies directed against Iba1 and GFAP to analyze microglia and astrocytes, respectively. No obvious morphological signs of glial activation were observed in the cerebral cortex of HSP60-treated mice (Figure 5A, D). Also, assessment of the mean fluorescence intensity of Iba1- and GFAP-positive cells revealed no significant differences between animals injected with HSP60 and control animals (Figure 5B, E). In line with these findings, quantification of Iba1- and GFAP-positive cells in the cerebral cortex revealed no differences in cell numbers between animals that received intrathecal HSP60 and animals treated with the control protein SA (Figure 5C, F). Likewise, differences in the morphology or number of CD11b-positive cells in the cerebral cortex of animals injected with SA and animals treated with HSP60 were not detected (data not shown).

To further investigate whether intrathecal HSP60 causes proliferation of cells in the cerebral cortex, brain sections of the injected animals were immunostained with the intrinsic proliferation marker Ki67 (Figure 5G). No Ki67 reactivity was detected in the cerebral cortex of HSP60-treated mice or animals injected with SA. However, Ki67-positive cells were present regularly in the subgranular zone of the hippocampal dentate gyrus, in which proliferation of neuronal precursors occurs, as expected.

### In comparison to LPS, intrathecal HSP60 does not provoke a robust inflammatory response in the CNS

**Figure 6:**
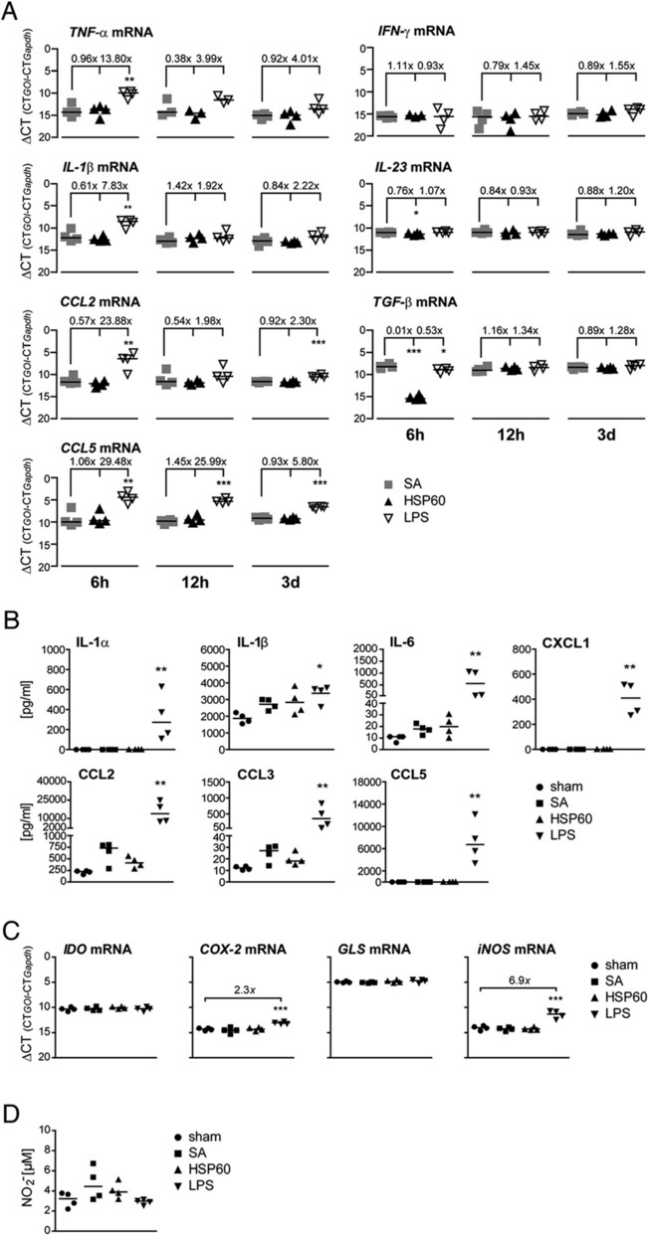
**Compared to LPS**, **intrathecal HSP60 does not induce considerable inflammation in the CNS. (A)** C57BL/6 J mice were injected intrathecally with 40 μg HSP60 (*n* = 4), 40 μg SA (control, *n* = 4), or 10 μg LPS (*n* = 4). After 6 h, 12 h, and 72 h brain lysates were analyzed for the expression of various genes encoding proinflammatory molecules, as indicated, by qPCR. Normalized values (ΔCT) per animal with the median of the respective group; *p < 0.05, **p < 0.005, ***p < 0.001; ANOVA of log2 transformation followed by Bonferroni-selected pairs; versus SA. Fold increase to SA was calculated with the median. **(B)** C57BL/6 J mice were injected intrathecally with 40 μg HSP60 (*n* = 4), 40 μg SA (control, *n* = 4), or 10 μg LPS (*n* = 4). Sham-operated animals served as a further control group (*n* = 4). After 12 h brain lysates were analyzed for the expression levels of proinflammatory proteins, as indicated, by multiple analyte detection and Il-1β ELISA. Median; *p < 0.05, **p < 0.005; Kruskal-Wallis followed by Dunn’s selected pairs; versus sham. **(C, D)** C57BL/6 J mice received sham surgery (*n* = 4) or were injected intrathecally with 40 μg HSP60 (*n* = 4), 40 μg SA (*n* = 4), or 10 μg LPS (*n* = 4). After 12 h brain lysates were analyzed for **(C)** mRNA expression of various enzymes associated with the production of neurotoxic metabolites, as indicated, and **(D)** the content of nitrite (NO). **(C)** Levels of mRNA expression were determined by qPCR and expressed as ΔCT per animal with the median; ***p < 0.001; ANOVA of log2 transformation followed by Bonferroni-selected pairs; versus sham. **(D)** Determination of the amount of NO by Griess’ reaction; Median, Kruskal-Wallis test followed by Dunn’s selected pairs.

Activation of TLRs expressed in glial cells by their cognate ligands typically results in a strong inflammatory response and peak in the expression of proinflammatory genes [25]. In order to determine HSP60’s capacity to induce inflammation in the CNS, mouse brains challenged with HSP60 were analyzed for the expression of proinflammatory mediators. To this end, C57BL/6 J mice were injected intrathecally with HSP60 or SA. Further control groups received sham surgery or were injected intrathecally with LPS as a positive control for TLR4 activation. After 6 h, 12 h, and 72 h, whole brain lysates were analyzed by quantitative real-time PCR using primers specific for genes encoding various proinflammatory cytokines and chemokines that are typically involved in the innate immune response [26,27], as indicated (Figure 6A). Whereas intrathecal injection of LPS resulted in a robust upregulation of most of the tested proinflammatory genes at at least one time point, intrathecal injection of HSP60 did not change the expression levels of the tested genes during the whole round of observation with the exception of TGF-β (0.01-fold, *p* < 0.001) and IL-23 (0.76-fold, *p* < 0.05), which were significantly downregulated compared to SA injection after 6 h (Figure 6A).

Analysis of protein content in brain lysates from mice injected with HSP60 was performed using the bead-based immunoassay FlowCytomix and ELISA. The results confirmed that, in contrast to LPS, intrathecal HSP60 did not cause significant changes in the content of proinflammatory mediators, such as IL-6, CXCL1, and CCL5, compared to control conditions (Figure 6B).

In the CNS, reactive oxygen species such as nitric oxide (NO) and further diverse neurotoxic metabolites contribute to neuronal and oligodendrocyte injury in inflammatory states [28,29]. To test whether intrathecal HSP60 and subsequent CNS injury are associated with the induction or accumulation of reactive oxygen species and neurotoxic metabolites, C56BL/6 J mice were injected intrathecally with HSP60. Control groups received sham surgery, SA, or LPS for comparative analysis. After 12 h, brain lysates were tested for mRNA expression of neurotoxic metabolite producing enzymes including indoleamine 2,3-dioxygenase (Ido), cyclooxygenase 2 (Cox-2), glutaminase (Gls), and inducible nitric oxide synthase (iNOS) by quantitative real-time PCR (Figure 6C), and were analyzed for NO content using the Griess reaction (Figure 6D). Neither mice injected with HSP60 nor animals injected with SA displayed changes in the expression levels of any of the tested candidate genes compared to sham animals. In contrast, intrathecal injection of LPS led to a 2.4-fold increase of *Cox-2* and a 7.4-fold increase of *iNo*s (both *p* < 0.005) in the brain compared to control conditions. However, no significant changes of NO content in the brain were detected in response to intrathecal HSP60 or LPS compared to control injections.

### Predominant expression of HSP60 in CNS neurons is upregulated in response to injury

**Figure 7:**
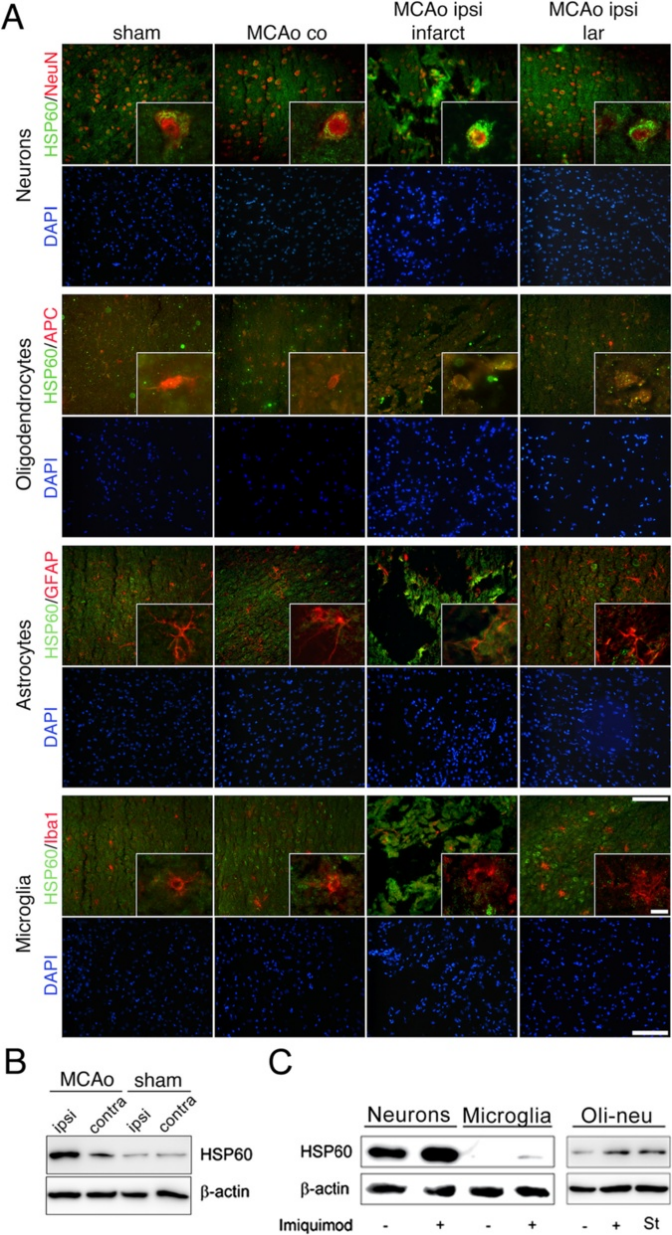
**HSP60 is predominantly expressed in CNS neurons and is upregulated after injury. (A)** Immunofluorescence photomicrographs of the infarct (ipsilateral; ipsi and contralateral; co) and lesion-associated region (lar) in brains of C57BL/6 J mice at 1 d after MCAo. Sham-operated animals served as a negative control. Tissues were double-stained with anti-Iba1, anti-GFAP, anti-APC and anti-NeuN antibodies to mark microglia, astrocytes, oligodendrocytes, and neurons, respectively, and with anti-HSP60. Nuclei were stained with DAPI. Scale bar, 100 μm. Cut-outs at higher power from the micrographs. Scale bar, 10 μm. **(B)** Ipsi- and contralateral hemispheres of C57BL/6 J mice at 1 d after MCAo and of the respective sham-operated animals were analyzed by SDS-PAGE and subsequent immunoblotting with an HSP60 antibody. β-actin served as a housekeeping control. **(C)** Lysates of cultured neurons and microglia, both incubated with 10 μg/ml imiquimod or PBS (control) for 12 h and 3 h, respectively, and lysates of Oli-neu cells incubated with 10 μg/ml imiquimod, 1 μM staurosporine (St), or PBS (control) for 3 h were analyzed by SDS-PAGE and subsequent immunoblotting with an HSP60 antibody. β-actin served as a housekeeping control.

Endogenous activators of the innate immunity such as HSP60 are evolutionary conserved molecules with the common feature of being foreign to the extracellular environment [30]. To determine the expression pattern of HSP60 in response to injury in the CNS, we made use of focal cerebral ischemia, a mouse model for stroke. Cerebral cortices of both stroked and sham-operated (control) mice were individually analyzed by immunohistochemistry using an antibody directed against HSP60 at 24 h after middle cerebral artery occlusion (MCAo). Although HSP60 is thought to be expressed ubiquitously [31], expression of HSP60 in the brains of sham-operated mice and in the contralateral unaffected hemispheres of animals treated with MCAo was detected predominantly in neurons (Figure 7A). In oligodendrocytes only weak or none immunoreactivity against HSP60 was observed. Neither microglia nor astrocytes displayed detectable HSP60 expression. In mice treated with MCAo expression of HSP60 in neurons and less pronounced in oligodendrocytes of the infarct area and the lesion-associated region of the ipsilateral brain hemisphere was clearly enhanced as compared to sham-operated animals and compared to the contralateral hemisphere of the MCAo-treated animals (Figure 7A). Stereological assessment of HSP60-positive objects and the percentage area of HSP60-positive region of interest (Additional file 2: Figure S2A, B) and analysis of the mean fluorescence intensity of HSP60 reactivity (Additional file 2: Figure S2C) confirmed these results. Expression of HSP60 was not detectable in microglia or astrocytes of MCAo-operated mice (Figure 7A). Analysis of brain lysates by SDS-PAGE revealed increased expression of HSP60 at one day after MCAo compared to sham-operated mice (Figure 7B), as expected [32,33]. To further explore the expression of HSP60 in CNS cells, lysates of cultured cortical neurons, microglia, and Oli-neu cells were analyzed by SDS-PAGE using an antibody directed against HSP60 (Figure 7C). While a pronounced and weak expression signal for HSP60 was detected in naïve neurons and oligodendrocyte precursors, respectively, no immunoreactivity was observed in preparations of naïve microglia. Expression of HSP60 was increased in neurons incubated with the TLR7 ligand imiquimod, which is known to induce cell-autonomous apoptosis in neurons [34], compared to unstimulated control conditions. Similarly, HSP60 expression was increased in Oli-neu cells incubated with imiquimod or the cytotoxin staurosporine, compared to untreated control conditions. In microglia, weak expression of HSP60 in response to imiquimod treatment was detected (Figure 7C).

These results show that HSP60 is predominantly expressed in neurons and that its expression is increased after injury.

### Endogenous HSP60 is released in CNS injury

**Figure 8:**
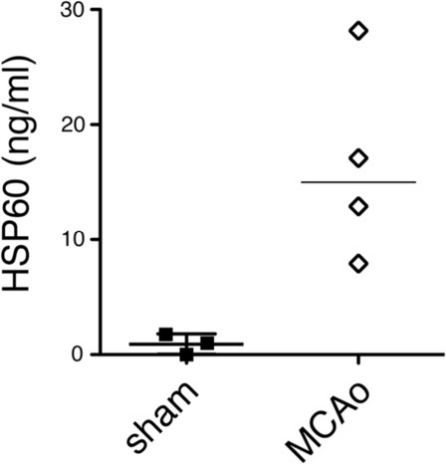
**CSF of stroked mice contains increased amounts of HSP60** **.** CSF from C57BL/6 J mice treated with focal cerebral ischemia (MCAo, *n* = 4) and from control subjects (sham, *n* = 3) was assayed by ELISA using an antibody directed against HSP60. Results are presented with the median. Statistical analysis was performed using the Mann–Whitney *U* test, *p* = 0.0651 for the comparison of both groups.

HSP60 predominantly localizes to mitochondria [31]. In the CNS, its intracellular expression is increased in response to various types of injury [35,36]. Along the same line, we have observed an increase of HSP60 expression in CNS neurons in response to ischemic injury *in vitro* and *in vivo*. For endogenous HSP60 to be capable of serving as an injury-related signal in the brain, it must exist extracellularly. Thus, we quantified levels of HSP60 protein in the CSF of mice treated with focal cerebral ischemia using ELISA and compared them to CSF of the sham-operated group (Figure 8). Whereas HSP60 protein was undetectable or present in only minute amounts in the CSF of control animals, the CSF of all mice treated with MCAo contained measurable and higher levels of HSP60. Although statistical significance was not reached within our experimental set-up, these results suggest that HSP60 is released during injurious processes in the CNS *in vivo*, thereby gaining the capability of serving as a danger signal in CNS injury.

## Discussion

Heat shock proteins released from damaged cells are key molecules during injury and inflammation and are capable of activating immunity. Accordingly, we showed in previous work that dying CNS cells release HSP60 *in vitro*, thereby leading to activation of microglia and subsequent neuronal injury [17]. In contrast to the ongoing intensive research on the HSP60’s effects on cells of the peripheral immune system, the biological impact of extracellular HSP60 on the CNS *in vivo* was undetermined so far. In the present study we demonstrate that intrathecal HSP60 mediates neurodegeneration and demyelination *in vivo*.

In cerebral ischemia, TLRs contribute to CNS injury [37,38] and their expression was detected mainly in activated glia [38]. These findings suggest that TLRs expressed in glia may recognize endogenous TLR ligands released during ischemic brain injury. However, the identity of such endogenous ligands is unknown. On the basis of our data it is tempting to speculate that HSP60 might be at least one of them. Our finding that HSP60 is overexpressed in injured neurons and oligodendrocytes and can be detected extracellularly in response to CNS injury match with the results from several other studies. For example, the amount of HSP60 in the serum of patients with a cerebral infarct is increased [39]. Also, soluble HSP60 was detected in the CSF of children suffering from traumatic brain injury [40].

At this stage, it remains unknown how HSP60 applied intrathecally exerts the observed degenerative effects in the brain. However, as described for other large proteins, intrathecal HSP60 may enter the brain tissue via receptor-mediated cellular uptake and intercellular transfer in principle [41]. Several studies have reported intrathecal delivery of large proteins to the brain parenchyma. Moreover, in CNS diseases including stroke and neurodegenerative lysosomal storage disorders intrathecal application of such proteins can result in deep penetration into the brain tissue and is functionally effective [42]. Also, we cannot rule out that intrathecal HSP60 leads to an inflammatory response that in turn causes degenerative effects in the brain paremchyma. Furthermore, it is unclear how much HSP60 is effectively released within the brain parenchyma at the site of injury in the context of CNS disorders and what the local concentration maxima are. The concentration of HSP60 used in the current study was based on our previous work *in vitro* and on our experience with a ligand for TLR2 *in vivo* [43,44], and must be considered supraphysiologic. Our study is a proof of principle regarding the effects of HSP60 in the brain, and future work will be required to determine key properties of HSP60 as a signaling molecule in the brain and the pathophysiological concentration of HSP60 that is involved in CNS damage.

Glial activation regularly occurs in the setting of neuronal injury and demyelination [24]. Microglia expressing TLR4 and releasing nitric oxide are required in neuronal injury induced by extracellular HSP60 *in vitro* [17]. However, although HSP60 activates the immune response in the periphery [45,46], results of our present study indicate that intrathecal HSP60 does not result in major activation or proliferation of glia and does not induce a considerable inflammatory response, as provoked by LPS, in the cerebral cortex *in vivo*. Notwithstanding, subtle activation of glial cells *in vivo* likely occurs, as these cells constantly monitor their microenvironment and respond to injury in their proximity [24]. We cannot rule out that specific inflammatory molecules different from the ones tested accumulate in response to intrathecal HSP60 or that the period of observation in our set-up was too short. Also, the expression of distinct proinflammatory mediators in the brain may vary depending on the concentrations of intrathecal HSP60, which we did not test. Although the release of inflammatory molecules from microglia activated by soluble danger signals has been demonstrated *in vitro* [17,47], the situation in the CNS *in vivo* is likely a lot more complex due to interaction of different cell types and further regulatory mechanisms. Indeed, recent studies on peripheral immune cells suggest that distinct inflammatory responses induced by HSP60 via TLR4 and TLR2 are dependent on the respective pathological context *in vivo* [48,49]. Also, mechanisms that operate directly through neurons and oligodendrocytes have to be taken into account. TLR3, TLR8, and TLR7 mediate cell-autonomous neuronal and axonal injury upon activation [44,50,51]. Although the expression of TLR4 in neurons is discussed controversely [52,53] and so far no significant expression of TLR4 in oligodendrocytes was detected [23], TLR4 may be up-regulated and activated in these cells in the presence of HSP60 *in vivo*. However, in this case yet unidentified factors that have an impact on TLR activation must exist, since neurons and oligodendrocytes alone are not affected by HSP60 *in vitro*. Also, it cannot be ruled out that the observed degenerative effects *in vivo* are not mediated by direct interaction between HSP60 and TLRs, but rather are caused by unknown secondary effects following the intrathecal application of HSP60.

It seems intriguing that activation of the same receptor and signaling molecule in the brain, namely TLR4 and MyD88, results in a different outcome regarding injury and inflammation depending on the ligand used. Intrathecal injection of the exogenous ligand LPS caused a robust inflammatory response but no CNS injury, while intrathecal injection of the endogenous ligand HSP60 led to neurodegeneration as well as demyelination but failed to induce a comparable inflammatory response in the brain as provoked by LPS. Although it is unknown, how inflammation and neuronal damage mediated by TLRs are related in the brain different pathways downstream of TLR4 and MyD88 may be activated in the CNS, depending on the ligand. Furthermore, taking into account the observed trend towards enhanced TUNEL+ positivity in brains of MyD88-deficient mice, but not in brains of TLR4-deficient animals after HSP60 treatment (see Figure 2D), we cannot rule out at this stage that other molecules downstream of TLR4 besides MyD88 contribute - at least in part - to HSP60-induced CNS injury. SARM, which is preferentially expressed in neurons and may play a crucial role in neuronal and axonal injury [54], is a possible candidate molecule.

At least in the periphery HSP60 triggers a multifaceted process, dependent among others, on its interaction with other receptors [55,56]. Also, TLR4 exerts not only detrimental but also regenerative effects upon injury [57]. The interaction between HSP60 and TLR4 may therefore not only cause damage in the CNS, but may also induce anti-inflammatory and neuroprotective effects [58,59].

Signaling by HSPs has long been the subject of considerable debate [45]. In particular it was discussed that the effects of HSP60 on macrophages were likely to be caused by LPS contaminating recombinant HSP60 preparations [60]. We showed previously that neurotoxic effects mediated by HSP60 are not caused by LPS [17]. Notably, we used a low-endotoxin HSP60 batch in our studies, and this preparation was used after dilution to such an extent that the theoretical maximal content of LPS was by far not sufficient to induce neuronal injury *in vitro* [17]. We show here that intrathecal LPS itself does not affect neuronal and oligodendrocyte survival in the cerebral cortex *-* in contrast to recombinant HSP60, which leads to degenerative processes *in vivo*.

Besides TLR4, other receptors are involved in HSP60-induced inflammatory effects. LOX-1 serves as a receptor for HSP60 in dendritic cells in lung inflammation [61]. The microglial TREM2 receptor was identified as an HSP60-binding protein, whose binding resulted in an increase in phagocytosis [62]. Finally, not only TLR4 but also TLR2 in human fibroblasts responds to HSP60 [2]. It is unknown whether the receptors named above are involved in CNS injury *in vivo*. Also, other endogenous ligands besides HSP60 are released from both injured peripheral and CNS cells. For example, β-defensin, and HSP70 activating TLR2 and TLR4 [3,6], but also ligands of other receptors, such as let-7 miRNA activating TLR7 [44] and HMGB1 binding to RAGE [63], are host-derived molecules involved in inflammation and tissue damage. It is conceivable that many more yet unidentified molecules are released during tissue injury. However, since the identity of such molecules and the associated biological processes are still poorly defined, one can only estimate at this stage to which extent their interaction is biologically relevant in a given CNS disorder.

## Conclusions

In summary, extracellular HSP60 mediates CNS injury *in vivo* through a TLR4- and MyD88-dependent pathway. On the basis of our previous results obtained *in vitro* and of our current studies *in vivo* we propose a molecular mechanism by which CNS injury leads to the release of HSP60, which in turn activates TLR4 signaling in the brain. As a consequence, further neurodegeneration and demyelination ensues. Both neurodegeneration and demyelination, which are common hallmarks of various CNS disorders, may be thus bi-directionally linked with an ancient host response system through conserved molecules such as the HSPs and innate immune receptors such as the TLRs.

## Material and methods

### Animals

C57BL/6 J mice were purchased from Charles River, Sulzbach, Germany. TLR4^−/−^ and MyD88^−/−^ mice were generously provided by Dr. S. Akira (Osaka University, Department of Host Defense, Osaka, Japan). All animals were maintained according to the guidelines of the committee for animal care. In detail, mice were housed in groups with chip bedding and environmental enrichment on a 12 h light/dark cycle with *ad libitum* access to water and food (standard chow). All experiments were conducted in accordance with the European directive on the protection of animals used for scientific purposes and approved by the institutional review committee Landesamt für Gesundheit und Soziales, Berlin, Germany (LAGeSo registration TVA G 0175/07).

### Primary culture of neurons and microglia

Purified microglia were generated from forebrains of neonatal P0-3 mice, as previously described [18]. Briefly, brain tissue was dissociated with trypsin (Invitrogen, Darmstadt, Germany) for 20 min at 37°C. After mechanical dissociation, cells were plated in 75-cm^2^ culture flasks in DMEM (Invitrogen, Darmstadt, Germany) supplemented with 10% FBS and penicillin/streptomycin. After 1 week in culture, mixed glial cultures were shaken for 30 min at 180 rpm. The supernatant containing >95% microglia was plated on poly-d-lysine-coated (BD Biosciences, San Jose, USA) glass coverslips. Microglia were maintained in DMEM with 10% FBS.

Primary cultures of cortical neurons were generated from forebrains of E17 mice. Cortices were dissociated by trituration with papain (Worthington, Lakewood, USA) in EBSS (Invitrogen, Darmstadt, Germany) for 5 min at 37°C. Subsequently, cells were resuspended in 0.25% trypsin inhibitor and 0.25% BSA (both obtained from Sigma-Aldrich, Munich, Germany) in EBSS, and incubated at 37°C for 5 min. Cells were pelleted by centrifugation at 1000 × *g* for 5 min. The cell concentration was adjusted to 1 × 10^6^ cells/ml in MEM with GlutaMAX medium (Invitrogen, Darmstadt, Germany) supplemented with 10% FBS and penicillin/streptomycin. A total of 5 × 10^5^ cells/24-well were plated onto poly-d-lysine-coated glass slides (BD Biosciences, San Jose, USA) and were maintained in humidified 5% CO_2_/95% air at 37°C. Immediate immunostaining revealed 90–95% purity for neurons.

At day 3 after plating of neurons half of the media per well was replaced by 60.000 microglia in DMEM. Co-cultures of microglia and neurons were incubated with the respective agents after a further 12 h.

### Oli-neu precursor cell line and co-cultures containing Oli-neu cells and microglia

Oli-neu cells were generously provided by Dr. J. Trotter (Institute of Molecular Biology, Johannes Gutenberg-University, Mainz, Germany) [64] and were cultured in DMEM (Invitrogen, Darmstadt, Germany) supplemented with 10% FBS and penicillin/streptomycin. At 12 h after plating of 6.000 Oli-neu cells half of the media per 24-well was replaced by 60.000 microglia in DMEM. Co-cultures of microglia and Oli-neu cells were incubated with the respective agents after a further 12 h.

### Intrathecal injection into mice

Intrathecal injection into mice was performed as described previously [43]. In brief, male C57BL/6, TLR4^−/−^, or MyD88^−/−^ mice (~20 g) were anesthetized with i.p. ketamine (100 mg/kg; AllemanPharma, Pfullingen, Germany) and xylazine (16 mg/kg; Bayer, Leverkusen, Germany). A skin incision was made to expose the lumbar spine. Using a 30-gauge needle and a microliter syringe, 40 μl of water alone, LPS, HSP60, or SA solution, as indicated, were slowly injected into the spinal canal at vertebrae L2 or L3. The skin incision was closed using dermal clips. Animals were then allowed to wake up and given free access to food and water. Absence of paresis and adequate waking were verified. For toxicity studies, 40 μg recombinant human HSP60-Low Endotoxin (Enzo Life Sciences, Lörrach, Germany) was injected. The contamination with LPS of the HSP60 preparation as declared by the manufacturer was <50 EU/mg and ≤1.67 EU/mg, as determined by an independent laboratory specializing in endotoxin testing and published in previous work (Mikrobiologisches Labor, Münster, Germany; see also [17]). Human serum albumin (SA, Ultra-Low endotoxin, HumanZyme, Chicago, USA, 40 μg) was used as control protein with a similar molecular weight as HSP60 (60 kDa). LPS (0111:B4) was purchased from Enzo Life Sciences, Lörrach, Germany.

### Model of experimental murine stroke

Stroke was induced by middle cerebral artery occlusion (MCAo) in C57BL/6 J male mice at 10–12 weeks of age, as described previously [65]. Briefly, under isoflurane (Abott, Wiesbaden, Germany) anaesthesia in a 1:2 mixture of oxygen/nitrous oxide a monofilament was inserted into the common carotid artery, advanced to the origin of the MCA, and left in place for 60 min until reperfusion. In sham-operated animals, the monofilament was also inserted and advanced to the MCA, but was withdrawn immediately, thereby avoiding ischemia. Mice were kept in heated cages for the next 2 h, and rectal temperature was measured regularly. Cerebrospinal fluid (CSF) samples were obtained, as previously described [43], after one day. In brief, a skin incision was made over the head and neck. After dissection of the suboccipital muscles, the cisterna magna was punctured and CSF withdrawn using a 27-gauge butterfly cannula connected to a microliter syringe. Brains were removed, snap frozen in methylbutane, and stored at −80°C until further processing.

### Immunocytochemistry and immunohistochemistry

Seventy-two hours after intrathecal injection or 24 h after MCAo, mice were deeply anesthetized with i.p. ketamine and xylazine and then fixed by transcardial perfusion with PBS followed by 4% paraformaldehyde (PFA) in phosphate buffered saline (PBS). Forebrains were post-fixed in 4% paraformaldehyde in PBS overnight, then cryoprotected in a row of 10%, 20%, and 30% sucrose. Cryostat coronal sections (15 μm) were thaw-mounted on coated glass slides. Representative brain sections (level 1: interaural 6.60 mm; level 2: 5.34 mm; level 3: 3.94 mm; level 4: 1.86 mm; level 5: −0.08 mm) or cell cultures were fixed with 4% PFA, washed with PBS, and treated with blocking solution (5% normal goat serum) for 1 h. They were then incubated with the respective primary antibody: anti-NeuN, anti-neurofilament (200 kDa, clone RT97), anti-myelin basic protein (MBP), anti-glial fibrillary acidic protein (GFAP), or anti-adenomatous polyposis coli (APC) (all purchased from Merck Millipore, Darmstadt, Germany), anti-HSP60 (Enzo Life Sciences, Lörrach, Germany), anti-Ki67 (ab 16667, Abcam, Cambridge, UK), anti-Iba1 (WAKO, Neuss, Germany), anti-CD11b (eBioscience, Frankfurt, Germany) or the isolectin IB4 (Invitrogen, Carlsbad, USA) overnight at 4°C. Subsequently, sections or cell cultures were incubated with the relevant secondary antibody (all purchased from Jackson Immuno Research, West Grove, USA) for 1 h at room temperature.

TUNEL staining of co-cultures and the CNS cortex was conducted after 3 d of incubation and 5 d after intrathecal injection, respectively, using the Apoptaq Plus Fluorescein In Situ Apoptosis Detection Kit, following the instruction manual (Merck Millipore, Darmstadt, Germany). DAPI staining was performed as previously described [18]. Immunofluorescence images were obtained using an Olympus BX51 microscope.

### Quantification of CNS cells in brain sections

Viability of neurons and oligodendrocytes in the brain’s cortex was analyzed by quantifying NeuN-positive and APC-positive cells, respectively, in six fields (x600) at level four of five representative sections of each brain. The mean was calculated, which is expressed as NeuN-positive or APC-positive cells per mm^2^, and each group is displayed with the median.

For analysis of apoptotic cells, brain sections were stained by TUNEL and DAPI. TUNEL-positive and DAPI-positive nuclei were counted in the cerebral cortex from representative levels one to five after visual verification of apoptotic hallmarks such as shrinkage and fragmentation, and the sum per field was calculated. Each group is displayed with the median.

Microglia and astrocytes were quantified by staining brain sections with anti-Iba1 and anti-GFAP antibodies, respectively. Iba1-positive or GFAP-positive cells were quantified in three fields per hemisphere at level four of five representative sections of each brain, and the mean was calculated, which is expressed as Iba1-positive or GFAP-positive cells per mm^2^. Each group is displayed with the median.

### Stereological analysis of brain sections

Stereological quantification of neurons and oligodendroglia in mice was performed using the CellSense^TM^ software provided by Olympus. For experiments involving intrathecal injections, six fields (×60) in the cortex of five representative brain sections stained with NeuN or APC antibody per animal were analyzed by quantifying NeuN-positive or APC-positive objects and determining the percentage area of NeuN-positive or APC-positive region of interest (ROI), as indicated. The threshold was set to 400 dpi (NeuN+) or 150 dpi (APC+). For analysis of TUNEL-stained cells, whole cortices of five representative brain sections per animal were analyzed by quantifying TUNEL-positive objects and the percentage of TUNEL-positive region of interest (×20, threshold 20 dpi).

### Measurement of fluorescence intensity in immunostained brain sections

For Iba1, GFAP, and MBP reactivity measures (mean fluorescence intensity per area) DAPI staining was used as reference. Mean fluorescence intensity of Iba1, GFAP, or MBP as assessed by Image J software was divided by the mean fluorescence intensity of DAPI from the same scan to assure technical accuracy. Representative brain slices containing the cortical area (20 μm) per animal were scanned using a confocal laser scanning microscope (Leica TCS SPE, Wetzlar, Germany). Z-stacks were analysed using Image J software by a person blind to the sample identity.

### Real-time PCR

One brain hemisphere was homogenized in 1 ml TRIzol® with an Ultra-Turrax® at 21500 rpm for 30 sec. The homogenate was centrifuged at 12000 x g and 4°C for 15 min. DNA was removed with RQ1 RNase-free DNase and UltraPure™ phenol:chloroform:isoamyl alcohol. For synthesis of cDNA from 1 μg RNA, random hexamers were used with MMLV-RT (Promega, Mannheim, Germany). SYBR® Green-based quantitative real-time PCR was performed with the RT^2^ qPCR Primer Assays (SABiosciences Corporation, Frederick, USA) according to the manufacturer’s manual with the RT^2^ Real-Time™ PCR protocol (#1) and the ABI7500 default dissociation stage. Glyceraldehyde-3-phosphate dehydrogenase (GAPDH) was used as a housekeeping gene for control. For statistics dCT values (CT_GOI_-CT_HKG_) were log2-transformed according to [66]. Fold exchange (2^-(ddCT)^) relative to the control was calculated using the median of each group. P values and stars indicate statistical significance, fold change values <0.5 and >2 indicate biological significance.

### Cytokine bead assay

Cytokine bead assay was performed with the FlowCytomix System (eBioscience, Frankfurt, Germany) according to the manufacturer’s manual. One half of a brain per mouse was lysed in 50 mM Tris pH 7.4 with cOmplete Ultra Tablet (Roche, Basel, Switzerland) with an Ultra-Turrax (5 section at 17500 rpm) on ice. Lysates were centrifuged at 10000 x g, 4°C for 30 min. Protein levels in supernatants were measured by BCA Protein Assay (Pierce, Rockford, USA).

### Analysis of NO content

The amount of nitric oxide in one brain hemisphere was determined by the standard Griess’ reaction, as previously described [17].

### HSP60 and Il-1β ELISA

Amounts of HSP60 in the CSF of mice and Il-1β in murine brain lysates were determined by ELISA according to the manufacturer’s respective manuals (Enzo Life Sciences, Lörrach, Germany, and BD Biosciences, Heidelberg, Germany, respectively).

### Western blot

Lysates (10 μg of protein) of primary neurons, microglia, and Oli-neu cells or lysates of brain hemispheres of MCAo-treated and sham-operated mice, as indicated, were applied to a 10% SDS-PAGE gel. HSP60 and neuronal nuclei were detected by immunoblotting using a mouse monoclonal antibody against HSP60 (Enzo Life Sciences, Lörrach, Germany) and neuronal nuclei (NeuN, Merck Millipore, Darmstadt, Germany), respectively. The mouse monoclonal antibody against synaptophysin was generously provided by Dr. M. Hoeltje (Charité-Universitaetsmedizin Berlin, Germany). Protein signals were visualized by enhanced chemiluminescence detection, as described previously [17], and intensities of the signals were quantified by Image J software.

### Statistical analysis

Data are expressed as indicated in the figure legends. Statistical differences between treatment groups were determined using Mann–Whitney U test, one-way and two-way ANOVA, or Kruskal Wallis test, as indicated. Differences were considered statistically significant when *p* < 0.05.

## Additional files

Additional file 1: Figure S1. Stereological analysis of neuronal and oligodendrocyte injury and loss in the cerebral cortex in response to intrathecal HSP60. (**A-D**) Forty micrograms of HSP60 or 40 μg of SA were injected intrathecally into 8-12 week-old C57BL/6J (WT, HSP60 *n*=11, SA *n*=8), TLR4^-/-^ (HSP60 *n*=11, SA *n*=8), and MyD88^-/-^ (HSP60 *n*=11, SA *n*=8) mice. Naïve mice received no injection (WT *n*=6, TLR4^-/-^ *n*=4, MyD88^-/-^ *n*=4). Injection of LPS into WT mice served as a positive control for TLR4 activation (*n*=10). Injection of water into WT mice served as a carrier control (*n*=7). After 3 d, the cerebral cortex was immunostained with NeuN antibody (**A**, **B**) or stained by TUNEL assay (**C**, **D**). Subsequently, stereological analysis was performed by (**A**, **C**) quantification of NeuN+ or TUNEL+ objects and (**B**, **D**) determination of the percentage of the area of NeuN+ or TUNEL+ ROI (region of interest). Results are presented as mean +/- SD, Mann-Whitney *U* test for indicated groups. (**E**, **F**) Three days after intrathecal injection of 40 μg HSP60 or 40 μg SA (control) coronal brain sections of C57BL/6J (WT, HSP60 *n*=4, SA *n*=4), TLR4^-/-^ (HSP60 *n*=4, SA *n*=4), and MyD88^-/-^ (HSP60 *n*=4, SA *n*=4) mice were immunostained with an antibody against APC. Subsequently, stereological analysis was performed by **(E)** quantification of APC+ objects and **(F)** determination of the percentage of the area of APC+ ROI. Results are presented as mean +/- SD, Mann-Whitney *U* test for indicated groups. Additional file 2: Figure S2. Stereological quantification and intensity analysis of HSP60 expression in the brain of mice treated with MCAo. Ipsilateral area of infarct (ipsi), respective contralateral area (co), and lesion-associated region (lar) in brains of C57BL/6J mice treated with MCAo (*n*=4) were immunostained after 1 d with an HSP60 antibody and with DAPI. Sham-operated animals served as a negative control (*n*=4). Subsequently, stereological analysis was performed by **(A)** quantification of HSP60+ objects and **(B)** determination of the percentage of the area of HSP60+ ROI (region of interest). **(C)** Analysis of mean HSP60+ fluorescence intensity in the brain regions of the animals named above. (**A**-**C**) Results are presented as mean +/- SD, Mann-Whitney *U* test for indicated groups.
