# Supplementary material for: Intrathecal heat shock protein 60 mediates neurodegeneration and demyelination in the CNS through a TLR4- and MyD88-dependent pathway
Source: Mol Neurodegener. 2015 Feb 26;10:5. doi: 10.1186/s13024-015-0003-1 (PMC4365525; doi:10.1186/s13024-015-0003-1)
Supplement: Additional file 1: Figure S1. — Stereological analysis of neuronal and oligodendrocyte injury and loss in the cerebral cortex in response to intrathecal HSP60. (A-D) Forty micrograms of HSP60 or 40 μg of SA were injected intrathecally into 8-12 week-old C57BL/6J (WT, HSP60 n=11, SA n=8), TLR4-/- (HSP60 n=11, SA n=8), and MyD88-/- (HSP60 n=11, SA n=8) mice. Naïve mice received no injection (WT n=6, TLR4-/- n=4, MyD88-/- n=4). Injection of LPS into WT mice served as a positive control for TLR4 activation (n=10). Injection of water into WT mice served as a carrier control (n=7). After 3 d, the cerebral cortex was immunostained with NeuN antibody (A, B) or stained by TUNEL assay (C, D). Subsequently, stereological analysis was performed by (A, C) quantification of NeuN+ or TUNEL+ objects and (B, D) determination of the percentage of the area of NeuN+ or TUNEL+ ROI (region of interest). Results are presented as mean +/- SD, Mann-Whitney U test for indicated groups. (E, F) Three days after intrathecal injection of 40 μg HSP60 or 40 μg SA (control) coronal brain sections of C57BL/6J (WT, HSP60 n=4, SA n=4), TLR4-/- (HSP60 n=4, SA n=4), and MyD88-/- (HSP60 n=4, SA n=4) mice were immunostained with an antibody against APC. Subsequently, stereological analysis was performed by (E) quantification of APC+ objects and (F) determination of the percentage of the area of APC+ ROI. Results are presented as mean +/- SD, Mann-Whitney U test for indicated groups. [file 13024_2015_3_MOESM1_ESM.pdf]

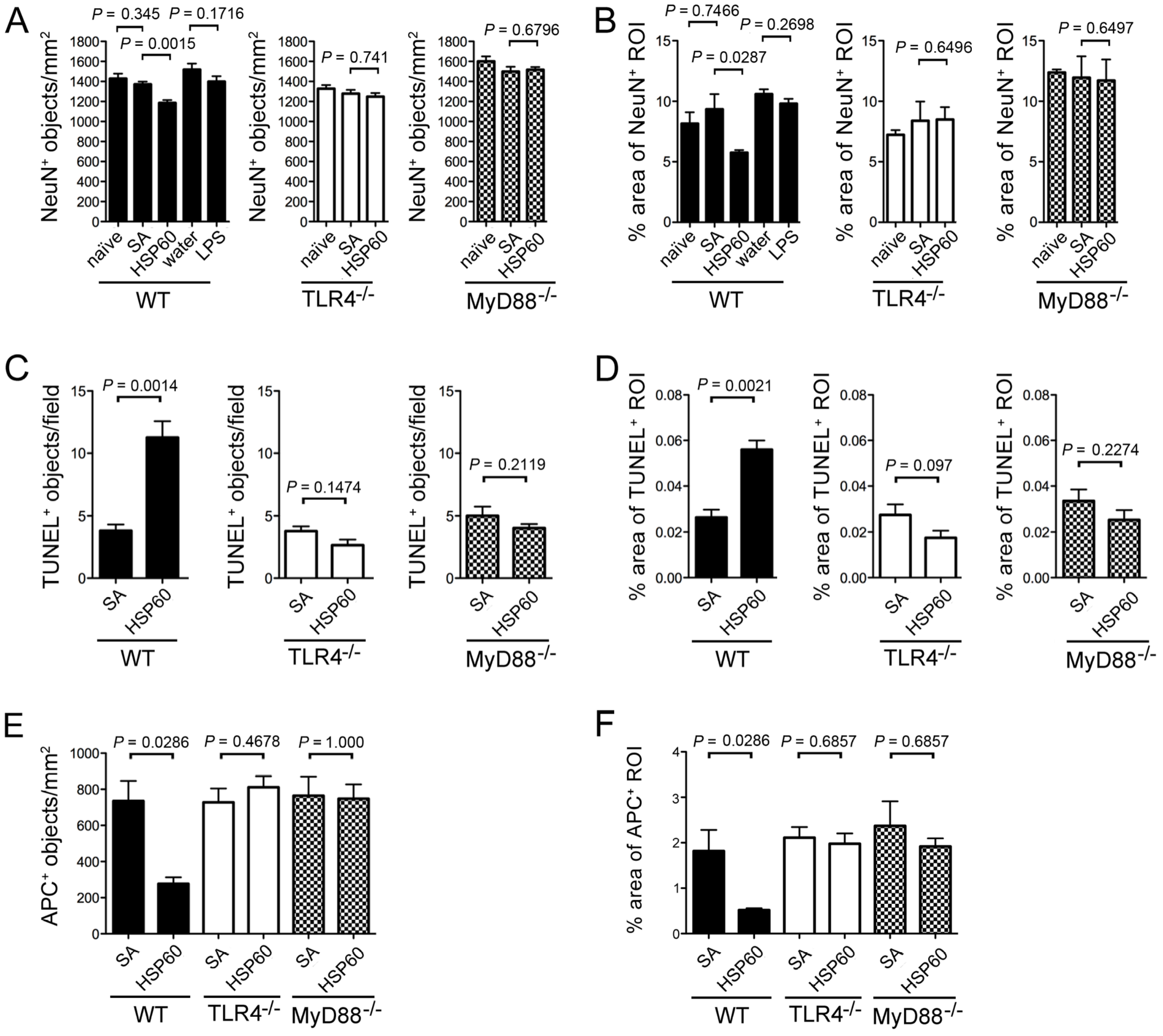

Supplementary Figure 1. Stereological analysis of neuronal and oligodendrocyte injury and loss in the cerebral cortex in response to intrathecal HSP60. (**A-D**) Forty micrograms of HSP60 or 40 μg of SA were injected intrathecally into 8-12 week-old C57BL/6J (WT, HSP60 *n*=11, SA *n*=8), TLR4$^{-/-}$ (HSP60 *n*=11, SA *n*=8), and MyD88$^{-/-}$ (HSP60 *n*=11, SA *n*=8) mice. Naïve mice received no injection (WT *n*=6, TLR4$^{-/-}$ *n*=4, MyD88$^{-/-}$ *n*=4). Injection of LPS into WT mice served as a positive control for TLR4 activation (*n*=10). Injection of water into WT mice served as a carrier control (*n*=7). After 3 d, the cerebral cortex was immunostained with NeuN antibody (**A**, **B**) or stained by TUNEL assay (**C**, **D**). Subsequently, stereological analysis was performed by (**A**, **C**) quantification of NeuN+ or TUNEL+ objects

and (**B**, **D**) determination of the percentage of the area of NeuN+ or TUNEL+ ROI (region of interest). Results are presented as mean +/- SD, Mann-Whitney *U* test for indicated groups. (**E**, **F**) Three days after intrathecal injection of 40 μg HSP60 or 40 μg SA (control) coronal brain sections of C57BL/6J (WT, HSP60 *n*=4, SA *n*=4), TLR4$^{-/-}$ (HSP60 *n*=4, SA *n*=4), and MyD88$^{-/-}$ (HSP60 *n*=4, SA *n*=4) mice were immunostained with an antibody against APC. Subsequently, stereological analysis was performed by (**E**) quantification of APC+ objects and (**F**) determination of the percentage of the area of APC+ ROI. Results are presented as mean +/- SD, Mann-Whitney *U* test for indicated groups.
