# Supplementary material for: Intrathecal heat shock protein 60 mediates neurodegeneration and demyelination in the CNS through a TLR4- and MyD88-dependent pathway
Source: Mol Neurodegener. 2015 Feb 26;10:5. doi: 10.1186/s13024-015-0003-1 (PMC4365525; doi:10.1186/s13024-015-0003-1)
Supplement: Additional file 2: Figure S2. — Stereological quantification and intensity analysis of HSP60 expression in the brain of mice treated with MCAo. Ipsilateral area of infarct (ipsi), respective contralateral area (co), and lesion-associated region (lar) in brains of C57BL/6J mice treated with MCAo (n=4) were immunostained after 1 d with an HSP60 antibody and with DAPI. Sham-operated animals served as a negative control (n=4). Subsequently, stereological analysis was performed by (A) quantification of HSP60+ objects and (B) determination of the percentage of the area of HSP60+ ROI (region of interest). (C) Analysis of mean HSP60+ fluorescence intensity in the brain regions of the animals named above. (A-C) Results are presented as mean +/- SD, Mann-Whitney U test for indicated groups. [file 13024_2015_3_MOESM2_ESM.pdf]

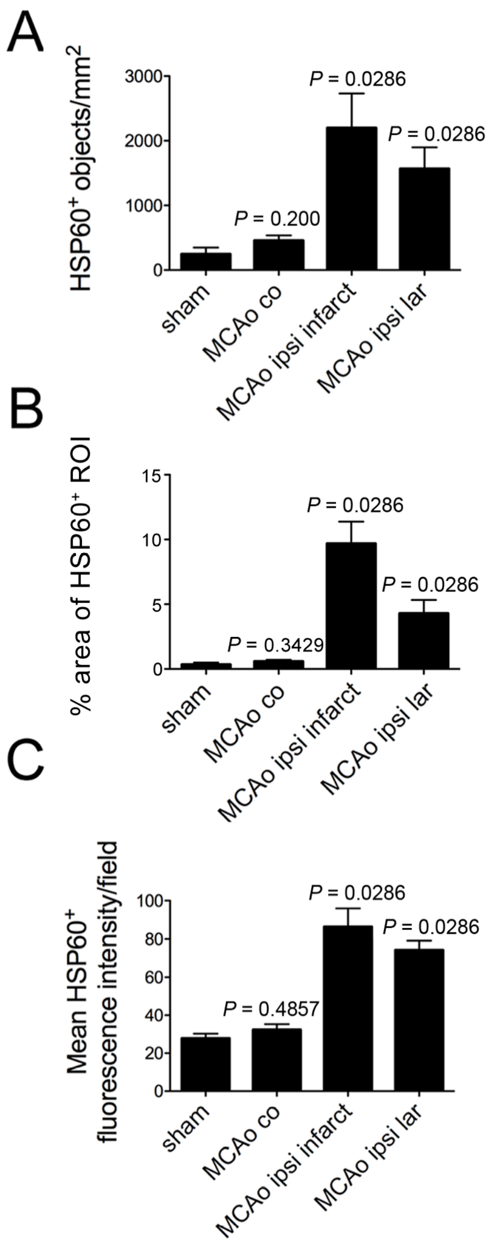

Supplementary Figure 2. Stereological quantification and intensity analysis of HSP60 expression in the brain of mice treated with MCAo. Ipsilateral area of infarct (ipsi), respective contralateral area (co), and lesion-associated region (lar) in brains of C57BL/6J mice treated with MCAo (n=4) were immunostained after 1 d with an HSP60 antibody and with DAPI. Sham-operated animals served as a negative control (n=4). Subsequently, stereological analysis was performed by (**A**) quantification of HSP60+ objects and (**B**) determination of the percentage of the area of HSP60+ ROI (region of interest). (**C**) Analysis

of mean HSP60+ fluorescence intensity in the brain regions of the animals named above. (**A-C**) Results are presented as mean +/- SD, Mann-Whitney *U* test for indicated groups.
